# Beyond the Cold: Activating Brown Adipose Tissue as an Approach to Combat Obesity

**DOI:** 10.3390/jcm13071973

**Published:** 2024-03-28

**Authors:** Cristina Elena Negroiu, Iulia Tudorașcu, Cristina Maria Bezna, Sanziana Godeanu, Marina Diaconu, Raluca Danoiu, Suzana Danoiu

**Affiliations:** 1 Department of Pathophysiology, University of Medicine and Pharmacy of Craiova, 200349 Craiova, Romania; bezna.mariacristina@gmail.com (C.M.B.); suzanadanoiu@yahoo.com (S.D.); 2 Doctoral School, University of Medicine and Pharmacy of Craiova, 200349 Craiova, Romania; sanzianagodeanu@yahoo.com; 3 Department of Physiology, University of Medicine and Pharmacy of Craiova, 200349 Craiova, Romania; 4 Department of Radiology, County Clinical Emergency Hospital of Craiova, 200642 Craiova, Romania; marina_trincu@yahoo.ro; 5 Department of Social Sciences and Humanities, University of Craiova, 200585 Craiova, Romania; danoiu.raluca.n8v@student.ucv.ro

**Keywords:** brown adipose tissue, obesity, browning agent, beige fat cell, FGF21

## Abstract

With a dramatic increase in the number of obese and overweight people, there is a great need for new anti-obesity therapies. With the discovery of the functionality of brown adipose tissue in adults and the observation of beige fat cells among white fat cells, scientists are looking for substances and methods to increase the activity of these cells. We aimed to describe how scientists have concluded that brown adipose tissue is also present and active in adults, to describe where in the human body these deposits of brown adipose tissue are, to summarize the origin of both brown fat cells and beige fat cells, and, last but not least, to list some of the substances and methods classified as BAT promotion agents with their benefits and side effects. We summarized these findings based on the original literature and reviews in the field, emphasizing the discovery, function, and origins of brown adipose tissue, BAT promotion agents, and batokines. Only studies written in English and with a satisfying rating were identified from electronic searches of PubMed.

## 1. Introduction

Thermogenesis, the biological process of heat generation, is essential for maintaining body temperature in warm-blooded animals. Brown adipose tissue (BAT) primarily contributes to nonshivering thermogenesis in mammals. BAT expresses the mitochondrial protein Uncoupling Protein 1 or UCP1, which facilitates a proton leak, uncoupling oxidative phosphorylation and increasing heat dissipation. This unique capability positions BAT as a key player in regulating global energy expenditure, making it a promising target for therapeutic interventions in obesity [1].

For many years, the prevailing belief was that BAT exhibited activity solely in newborns. It was widely believed that this tissue became inactive in adult humans. The dismissal of active metabolic BAT in adult humans became a medical dogma, although the exact evidence supporting this conclusion remains elusive. Notably, the role of BAT in cold adaptation metabolism in human adults has been vastly discounted. However, the advent of positron emission tomography (PET) in monitoring patients with neoplastic diseases has cast doubt on this entrenched belief [2].

In addition to its role in thermogenesis, recent research has highlighted the significance of BAT in regulating obesity. BAT promotion agents, such as cold exposure, β agonists, physical exercise, and dietary factors like green tea catechins and capsaicinoids, have been shown to stimulate BAT activity and promote energy expenditure. Furthermore, BAT secretes various batokines, including Interleukin 6 (IL6), Growth Differentiation Factor 15 (GDF15), Neuregulin4 (NRG4), and C-X-C motif chemokine ligand-14 (CXCL14), which play crucial roles in metabolic regulation and adipose tissue function. This comprehensive understanding of BAT’s role in obesity regulation underscores its potential as a therapeutic target and emphasizes the importance of further research in this field.

## 2. Discovery of Brown Adipose Tissue Activity in Adults through PET/CT Imaging

Using positron emission tomography–computed tomography (PET/CT) and the marker 2-18F-fluoro-2-deoxy-glucose (FDG), the activity of BAT has been discovered in the adult human body. At first, FDG uptake in the neck and shoulder areas was misinterpreted as a signal coming from the muscular tissue. With the addition of computed tomography (CT), the tissue density in these areas resembled adipose tissue, supporting the hypothesis that it was indeed BAT, thus reshaping our understanding of the presence and function of BAT in the adult human body [3,4,5].

A recent retrospective analysis of 134,529 positron emission tomography–computed tomography scans from 52,487 patients [6] shows that individuals with BAT exhibit lower prevalences of cardiometabolic diseases, independently correlating with reduced odds of type 2 diabetes, dyslipidemia, coronary artery disease, cerebrovascular disease, congestive heart failure, and hypertension. Individuals with overweight or obesity may benefit from BAT to promote overall cardiometabolic health.

## 3. Location of BAT

### 3.1. BAT Depots in Mice

Brown adipocytes are concentrated in specific BAT depots in mice, the primary model organism for brown fat studies [7]. These depots, including interscapular, subscapular, and cervical regions, exhibit variations in size and composition based on age, gender, and mouse strain background [8]. Noteworthy are additional small BAT depots proximal to major blood vessels and organs, such as periaortic and perirenal depots [8]. Recent imaging studies using 18F-FDG PET/CT and (123/125I)-β-methyl-p-iodophenyl-pentadecanoic acid with SPECT/CT suggest additional small pockets of cold-responsive fat depots in suprascapular, supraspinal, infrascapular, and ventral spinal regions [9].

### 3.2. Location of BAT in Human Adults

As mentioned earlier, PET/CT is a useful tool for identifying the presence of BAT, which is most abundant in the neck and supraclavicular region of adults. In addition to these two locations, BAT can be found in the paravertebral area; in the mediastinum, especially in the para-aortic area; around the heart, especially at the apex; and in infra-diaphragmatic depots, particularly in the perirenal space [10,11]. Obtaining accurate anatomical measurements of BAT is, however, difficult because it is distributed along with WAT in the neck, chest, and abdomen [12].

## 4. An Evolutionary Perspective on BAT

Our ancestors frequently faced extreme conditions in terms of cold or hunger over the years. BAT can be considered a survival organ, allowing defense against cold, minimizing shivering, and, therefore, enabling more efficient hunting in a hypothermic environment [13]. Modernization has allowed people to move indoors, with hypothermia being an uncommon threat. However, active BAT may represent the modern man as an intrinsic glycemic and lipidic buffering system.

## 5. Physiology

The involvement of the three β-adrenergic receptor subtypes (β1–3-AR) in thermogenic regulation exhibits species-dependent characteristics. In rodents, the β3-AR stands out as the most potent initiator of thermogenesis, as demonstrated by previous studies [14,15]. However, recent research on human BAT biopsies revealed the predominance of the β2-AR subtype, emerging as the primary driver of thermogenesis in brown adipocyte cell cultures derived from humans [16]. Intriguingly, silencing the β3-AR in cultured human brown adipocytes did lead to a reduction in their thermogenic activity [17], suggesting a potential role for both β2 and β3-AR subtypes in the thermogenic regulation of human brown adipocytes [18]. Furthermore, studies have indicated that noradrenaline-induced transcription primarily induces β2-AR, not β1-AR, in cultured human brown adipocytes, reinforcing the idea that both the β2 and β3-AR subtypes contribute to thermogenic regulation, with β2-AR likely playing a dominant role in human brown adipocytes.

**Figure 1:**
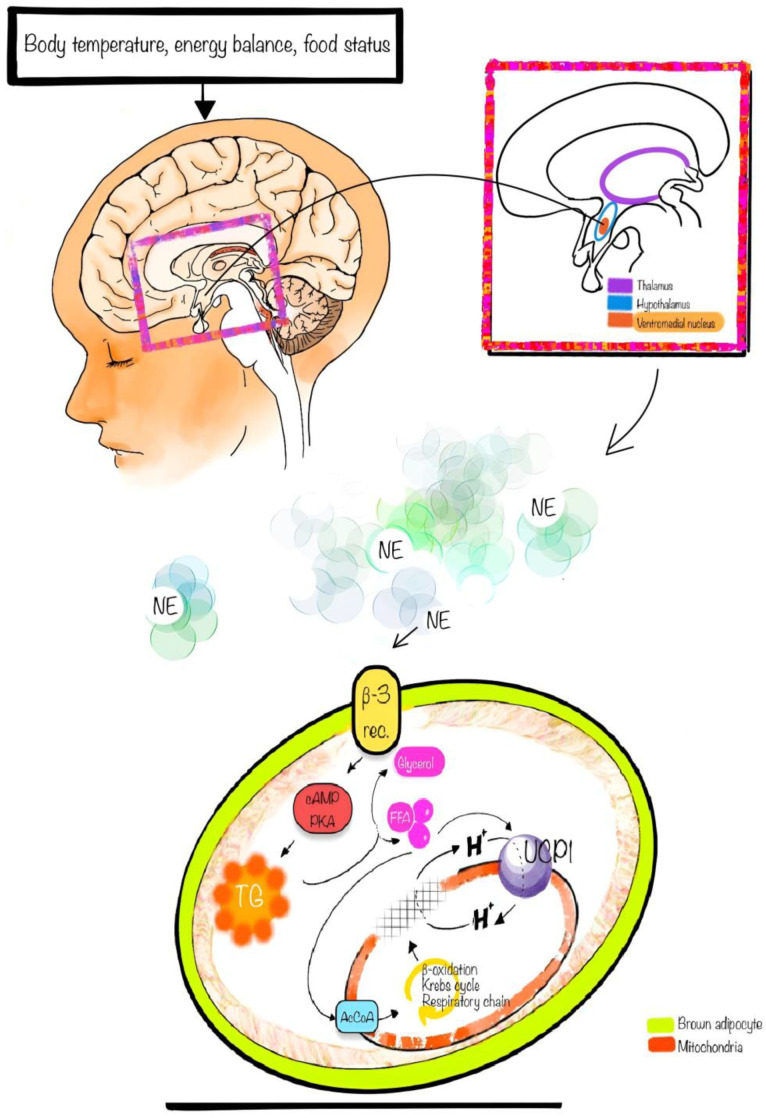
The relationship between the nervous system, norepinephrine, and brown adipocytes. Noradrenaline release initiates triglyceride degradation into fatty acids and glycerol by acting on the beta-3 adrenergic receptor. In the mitochondrial catabolism of fatty acids through beta-oxidation, the Krebs cycle and the respiratory chain create a potential across the mitochondrial membrane. A high concentration of hydrogen protons in the intermembrane space of mitochondria leads to the activation of UCP1. cAMP, cyclic Adenosine Monophosphate; PKA, protein kinase A; TG, triglycerides; AcCoA, acetyl-coenzyme A; UCP1, Uncoupling Protein 1; β3-rec, β3-receptor; FFA, free fatty acids.

Sympathetic nervous stimulation, prompted by environmental cues, triggers the release of norepinephrine, which binds to β-adrenergic receptors on the surface of brown adipocytes. The ensuing elevation in cAMP levels, resulting from β-receptor stimulation, facilitates the activation of protein kinase A (PKA). PKA, in turn, phosphorylates downstream targets, including p38 mitogen-activated protein kinase (MAPK), cAMP response element-binding protein (CREB), and hormone-sensitive lipase (HSL). The phosphorylation of p38 and CREB initiates the activation of genes involved in thermoregulation [19]. Simultaneously, the phosphorylation of HSL promotes lipolysis, liberating fatty acids that bind and directly activate UCP1 in thermogenic adipocytes [20]. Notably, lipolysis within BAT itself is not deemed obligatory for thermogenesis. These free fatty acids, both thermogenic substrates and UCP1 modulators, contribute to increased UCP1 activity (Figure 1). The catabolism of fatty acids through beta-oxidation, the Krebs cycle, and the respiratory chain generates a potential across the mitochondrial membrane. A high concentration of hydrogen protons in the intermembrane space of mitochondria activates UCP1, which serves as a symporter for protons and generates heat, in contrast to ATP synthase, which produces ATP [21]. Thus, brown adipose tissue’s thermogenic capacity involves utilizing glucose and fatty acids as precursors for heat generation.

## 6. Is UCP1 Indispensable for Thermogenesis?

The conclusive findings from mouse studies [22] revealed a vital role of UCP1 in thermogenesis. Oxygen consumption ratios between normal mice and those lacking UCP1 expression were compared, indicating significantly reduced oxygen consumption and inactivated thermogenesis in the absence of UCP1. The study showcased the impressive responsiveness of brown adipocytes to noradrenergic stimulation, leading to a tenfold increase in oxygen consumption compared to basal conditions. This response was notably absent in mice lacking the UCP1 protein.

However, the observation that mice lacking UCP1 die from hypothermia when suddenly exposed to cold temperatures but survive if the ambient temperature is gradually lowered suggests the existence of UCP1-independent thermogenic mechanisms [23]. This observation indicates that, in the absence of UCP1, the organism activates alternative mechanisms to maintain heat production and prevent hypothermia in cold conditions [24,25]. Nevertheless, these alternative mechanisms do not provide the same efficiency and thermal protection as the presence of UCP1.

The activation of lipolysis in BAT is not the exclusive requirement for thermogenesis. Recent data propose that brown adipose tissue predominantly relies on blood glucose and circulating free fatty acids released from the lipolysis of WAT as primary substrates for thermogenesis [7]. The crucial and open question is how brown adipocytes precisely select and utilize fuel [7].

## 7. Beige Fat Cells

In 2007 [2], it became clear that BAT also exists in adults, and more than that, it is also a functional tissue, but things did not stay that way for a long time. Since 2013, more studies [26,27] have highlighted other types of fat cells, which, despite morphologically and functionally resembling brown fat cells, are genetically more related to white fat cells. Unlike brown fat cells, which are located in distinct areas, beige fat cells are located in the white fat depots. Table 1 shows the characteristics of fat cell types.

Interestingly, with many therapeutic applications, beige fat cells are recruitable [28]. The induction of beige adipocytes has been demonstrated to occur under exercise, cold exposure, β agonists, physical exercise, BMP-7, and prostaglandins [1]. Similar to brown fat cells, beige fat cells are also characterized by the presence of many drops of lipids, a large number of mitochondria and β3 receptors, and the presence of genes specific to brown adipose tissue, such as UCP1, CIDEA (cell death-inducing DFFA-like effector A), and PGC1α (peroxisome proliferator-activated receptor-gamma coactivator) [1].

Both brown and beige adipocyte cells have the unique ability to transform the energy produced by the cell into heat, owing to the presence of the UCP1 protein. The ability of UCP1 to generate heat is similar to how ATP synthetase uses energy to create ATP.

Beige adipose tissue has attracted the attention of researchers because it poses two important questions: how do environmental factors influence cell differentiation, and how can we determine the transformation of white adipose tissue into beige adipose tissue?

## 8. Origins of Brown and Beige Cells

The mesoderm is considered to be the place where several specialized cell types develop, including myocytes, adipocytes, and chondrocytes. Most studies have concluded that BAT has a different origin from WAT [29,30]. Skeletal muscle and BAT have a common development; cells and their progeny have the capacity to activate the Myf5 (Myogenic factor 5) promoter [31]. Despite its lack of contractility, BAT has more in common with skeletal muscle tissue than WAT. Both are more specialized in lipid catabolism than in lipid storage, are innervated by the sympathetic nervous system, contain a large number of mitochondria, and play a role in adaptive thermogenesis [32].

In cultures, the presence of the transcription factor PRDM16 (PR Domain-Containing 16) led to the transformation of myoblasts into brown adipose tissue; conversely, the knockdown of PRDM16 in primary brown adipocytes induced myogenesis [33]. PRDM16 stimulates brown adipogenesis by binding PPAR-γ (peroxisome proliferator-activated receptor γ) and activating its transcriptional function. In addition, Bone Morphogenetic Protein-7 (BMP7) specifically directs brown adipocyte differentiation, including induction of PRDM16 and UCP1 gene expression [34].

It is now well known that there are two types of brown fat cells: classical brown fat cells, some with a positive Myf5 marker, and beige fat cells located between white fat cells. Beige fat cells are induced by β3 adrenergic stimulation, cold exposure, and exercise, are negative for the marker Myf5, and probably stem from the activation of dormant precursor cells [25,33]. Despite their different embryonic origins, beige fat cells express genes specific to brown adipose tissue, such as CIDEA, PGC1α, and UCP1 [35]. Many studies have attempted to identify pharmacological ways to recruit more beige fat cells to fight obesity.

## 9. Therapeutic Potential of BAT: BAT Promotion Agents

Due to its role in regulating energy homeostasis, the ongoing exploration of BAT has underscored its therapeutic potential in addressing the global obesity epidemic. Currently, few pharmacological agents are available for the treatment of obesity, and they have many side effects. With these therapies, weight loss is approximately 3% to 9% compared to placebo, and this weight loss is not maintained for a long time [36].

With the discovery of the functionality of BAT in adults and the observation of beige fat cells among white fat cells, scientists are searching for substances and methods to increase the activity of these cells. In the following lines, we will try to enumerate only a few substances and methods discussed as activators of brown adipose tissue.

### 9.1. Cold Exposure

Cold exposure was the most plausible activator of BAT. Cold exposure, whether in short durations of 2 h at temperatures between 16 and 18 °C [37,38,39] or extended periods of 5–8 h [40], as well as chronic exposure [10,41], stimulates the sympathetic nervous system (SNS), leading to the release of norepinephrine that acts on β3 receptors in BAT [42]. This physiological response not only holds therapeutic potential, such as treating depression by releasing norepinephrine and secreting endorphins [43], but also has implications for cardiovascular health. The prevalence of hypertension tends to increase in winter and cold regions, with cold temperatures exacerbating hypertension and triggering cardiovascular complications like stroke, myocardial infarction, and heart failure [42]. While this method may cause discomfort to the patient, repeated exposures to low temperatures are believed to potentially increase the frequency of respiratory infections. It is worth noting that adverse effects of prolonged hypothermia have been documented [44,45], but brief cold showers may not have sustained adverse impacts due to the remarkable efficiency of the thermoregulatory system in healthy individuals and animals [46]. To date, cold exposure has proven to be the most effective strategy for activating brown adipose activation, as noted above, with its side effects [47].

### 9.2. β-Adrenergic Receptor Agonists

β-adrenergic receptor agonists will be the next target of future studies because norepinephrine acts on these receptors in BAT to stimulate thermogenesis and lipolysis. Different molecules have been used in studies in mice but without proven action in humans [48]. Mirabegron is a drug used to treat overactive bladder and can be used at high doses to activate BAT [49]. Mirabegron, a β3 adrenergic receptor agonist, leads to an increase in blood pressure and pulse rate, primarily mediated through β1 adrenoreceptor stimulation [50]. While doses exceeding 150 mg daily showed adverse cardiovascular effects, 100 mg per day for four days demonstrated elevated energy expenditure without a concurrent rise in blood pressure or heart rate [51]. Notably, a 12-week treatment of obese, insulin-resistant men with 50 mg mirabegron per day yielded improvements in glucose homeostasis. This treatment induced the expression of thermogenic markers such as UCP1, TMEM26 (Transmembrane protein 26), and CIDEA in subcutaneous WAT, although it did not increase BAT volume based on PET/CT imaging [52]. The complexity of the signaling pathways involved in BAT activation and the interplay between different adrenergic receptors in humans necessitate further investigation to understand the nuanced effects of mirabegron and other β3-adrenergic receptor agonists on BAT. Despite the historical emphasis on the β3-AR agonist mirabegron as a potential pharmacological strategy for targeting human BAT, its poor performance in clinical trials and induction of cardiovascular responses have prompted alternative explanations. The data suggest that human BAT thermogenesis is primarily driven by β2-AR-mediated signaling, challenging the earlier hypothesis of β3-AR involvement and offering an alternative perspective on using mirabegron in eliciting BAT activation in humans [16].

### 9.3. Physical Exercise

Physical exercise emerges as a potent activator of BAT through its ability to enhance SNS activities and induce the secretion of irisin, FGF21, and cardiac natriuretic peptides [53,54,55]. This multifaceted response to exercise contributes to increased insulin sensitivity, improved glucose tolerance, and reduced circulating lipids [56,57,58]. However, caution is advised as exercise-associated side effects may manifest in individuals with specific pathologies, including severe hypertension, coronary heart disease, dyspnea at rest, and aortic stenosis [59]. Conflicting data on the impact of exercise on BAT activity are reported, with some studies indicating an increase [60,61], while others suggest a decrease in its activity [62,63,64].

### 9.4. 12,13-diHOME

12,13-diHOME, a lipokine secreted by BAT in response to cold exposure and physical exercise, plays a crucial role in regulating BAT fuel uptake, supporting thermogenic functions, and enhancing cardiac function and cardiomyocyte respiration [60,65,66]. Notably, it exhibits a strong negative association with BMI, triglyceride levels, and liver enzymes, highlighting its potential metabolic benefits [67,68]. Furthermore, 12,13-diHOME acts as an endocrine factor, activating fatty acid uptake and oxidation in skeletal muscle and contributing to improved fuel reserve in this tissue [60]. In addition to 12,13-diHOME, lipids such as omega-3 fatty acids and their precursors, like stearidonic acid and EPA (eicosapentaenoic acid), have been implicated in brown adipose tissue (BAT) activation and metabolic regulation [69].

### 9.5. Capsaicinoids

Capsaicinoids, acting as ligands for TRPV1 receptors, initiate the activation of Transient Receptor Potential Vanilloid channels 1 (TRPV1s), leading to the release of noradrenaline by the central nervous system [47,70]. They also play a role in modulating the expression of PPARγ coactivator α, facilitating the PPARγ-PRDM16 interaction, and promoting the activity of sirtuin-1 (SirT1) [70]. Beyond providing analgesic effects as an alternative for pain management, capsaicinoids offer protective benefits against cardiovascular disease, enhance insulin sensitivity, and demonstrate potential anti-cancer activity in lung, prostate, and breast cancers [71]. Although believed to induce both thermal and burning sensations upon contact with oral or skin mucosa, their co-carcinogenic effects in skin cancer necessitate further investigation [71,72].

### 9.6. Green Tea Catechins

Green tea catechins, with their thermogenic properties, primarily interact with caffeine to influence the sympathetic release of noradrenaline, contributing to its thermogenic effects [73]. Regular consumption of green tea is associated with various health benefits, including reduced blood pressure, improved diabetes management, weight reduction, and regulation of dyslipidemia [74]. Additionally, green tea has been shown to enhance cognitive functions and memory and exhibit anti-cancer properties by decreasing tumor growth factors and angiogenesis, promoting apoptosis in cancer cells, and reducing inflammation, anxiety, and stress [75]. While consuming up to three cups a day is generally well tolerated without significant adverse effects, caution is advised, especially on an empty stomach, as high doses of tea polyphenols may have a potentially toxic effect on the liver [76]. Studies recommend a daily intake of 3–4 cups of strong green tea containing 600–900 mg of catechins for at least eight weeks to experience optimal benefits, including reduced blood pressure, improved diabetes management, weight reduction, and regulation of dyslipidemia [77].

### 9.7. Fibroblast Growth Factor 21 (FGF21)

Fibroblast Growth Factor 21 (FGF21) is a well-studied batokine, primarily produced by the liver and secreted by white and brown adipose tissue [78,79]. It enhances the expression of thermogenic genes like UCP1 and DIO2 (stromal iodothyronine deiodinase 2), improving insulin sensitivity and having beneficial effects on body weight while lowering blood glucose and lipid levels [80,81]. However, chronic exposure to FGF21 has been associated with severe bone loss and growth retardation in mice due to the development of growth hormone resistance [82,83]. Overexpression of FGF21 has been shown to induce infertility in female mice [84]. Despite its positive effects on metabolism, further clinical trials are essential to understand potential side effects [85]. In subjects with type 2 diabetes, clinical studies demonstrated that administering an FGF21 analog significantly improved dyslipidemia. This improvement included reductions in plasma LDL cholesterol and triglycerides and an increase in HDL cholesterol. However, the anticipated glucose-lowering effect did not achieve statistical significance [81,86]. Based on an analysis by Chen [87], who evaluated 33 clinical trials with histological data, FGF21 analogs emerged as the top-ranked molecules for treating steatohepatitis. The involvement of BAT in mediating the favorable effects of FGF21 in humans requires further investigation.

### 9.8. Vascular Endothelial Growth Factor A (VEGFA)

Vascular Endothelial Growth Factor A (VEGFA) is a growth factor known for its role in angiogenesis and promoting vascular growth, proliferation, and migration [88]. Functioning as a batokine, VEGFA acts in a paracrine manner to modulate vascularization and induce thermogenesis in BAT [38,89,90], leading to the upregulation of UCP-1 and PGC-1 [91]. Overexpressing VEGFA in BAT increases thermogenesis in mice exposed to chronic cold and partially improves metabolic dysfunction associated with diet-induced obesity [92]. While VEGFA promotes angiogenesis, its proangiogenic role does not always confer benefits. Despite its positive impact on browning in WAT, its use in treating obesity-related disorders is limited due to the application of anti-VEGFA therapies for conditions like cancer and eye diseases [92].

### 9.9. Thiazolidinediones (TZDs)

Thiazolidinediones (TZDs), commonly used as insulin sensitizers for treating type 2 diabetes, act as PPAR-γ agonists, stimulating the expression of thermogenesis-specific genes in both brown and white adipose tissue [92,93,94]. While they effectively enhance insulin sensitivity, their use is associated with adverse effects such as heart failure, edema, weight gain, and bone loss [95,96].

### 9.10. Thyroid Hormones

Thyroid hormones, crucial regulators of metabolism and energy balance, have demonstrated the ability to induce WAT browning, a process augmented by sympathetic innervation [97,98]. Beyond influencing BAT activity, thyroid hormones impact BAT volume during the embryonic development of BAT in rats [99]. Besides their metabolic benefits, thyroid hormones play vital roles in controlling growth, lung function, heart function, and skeletal muscle development. However, their use may lead to heart-related issues, hyperthermia, and weight loss [100,101]. Despite insights from mouse and primate models, human clinical trials on thyroid hormone receptor stimulation remain scarce [102].

### 9.11. Bone Morphogenetic Protein-7 (BMP-7)

Bone Morphogenetic Protein-7 (BMP-7), belonging to the transforming growth factor-β superfamily, plays a crucial role in the formation of bone, kidneys, and BAT [103]. BMP-7 promotes the differentiation of mesenchymal progenitor cells into a brown adipocyte lineage, resulting in reduced fat mass, lower plasma glucose, and decreased plasma triglycerides [34]. Additionally, BMP-7 contributes to bone formation and healing [104]. However, its use may be associated with swelling, seroma, and an increased risk of cancer [105]. Despite potential side effects, BMP-7 has received FDA approval for clinical applications in long bone trauma, spinal fusion, and oral and maxillofacial procedures [106].

### 9.12. Glucagon-like Peptide-1 Receptor

Glucagon-like peptide-1 receptor (GLP-1R) agonists, including exendin, liraglutide, and semaglutide, have been shown to upregulate UCP-1 protein levels in BAT [107]. These agonists increase insulin and decrease glucagon secretion in a glucose-dependent manner, contributing to improved glucose regulation [107]. Additionally, GLP-1R agonists slow gastric emptying [108,109] and reduce appetite, making them beneficial for weight management [107]. However, common side effects such as nausea, vomiting, and diarrhea have been reported with their use [110]. The FDA approved the first GLP-1R agonist, exenatide, for the treatment of type 2 diabetes in 2005, and subsequent approvals have expanded their role in diabetes management [111].

The above examples demonstrate the variability of molecules and events that can activate BAT. The most important results are summarized in Table 2. In the literature, several reviews describe various BAT promotion agents [1,28,65,92]. These reviews state that the list of potential BAT promotion agents is considerable.

## 10. Experimental Approaches Used to Recognize the Endocrine Role of BAT

In the early days of endocrinology, ablation of a gland was often used to study its role [112]. This is not possible in the case of BAT due to the fact that the pathophysiological consequences related to its thermogenic or endocrinological activity could not be distinguished and due to the fact that BAT is diffusely localized. In rats, ablation of interscapular and axillary-localized BAT, which accounts for 40% of total BAT, is followed by compensatory expansion of other unresected tissues within a few days [113,114]. However, surgical ablation of BAT has resulted in varying degrees of body weight gain (or, in some cases, no change) and changes in glucose or lipid homeostasis [115,116].

Recently, surgical removal of BAT, in addition to genetic selection models, has been used to demonstrate that BAT is an endocrine source of myostatin [117] and 12,13-diHOME [60], which target skeletal muscle.

Brown fat plays an endocrine role in brown fat transplantation. Several studies have shown that transplantation of small amounts of BAT into obese and insulin-resistant rodent models results in increased insulin sensitivity, weight loss, and other metabolic improvements [118,119,120]. Transplantation of BAT reverses diabetes and improves glucose metabolism, which is attributed to an increase in IGF-1 levels [121].

## 11. Batokines

Similar to white adipose tissue, BAT secretes several endocrine, paracrine, or autocrine substances. These substances are called batokines, a few of which are also adipokines [14,122]. For example, adiponectin secreted by WAT is an important batokine, whereas leptin is secreted at a lower level in BAT than in WAT [11]

FGF 21 was one of the first batokines characterized and has attracted attention because of its pleiotropic effects and potential to treat metabolic diseases [78,79,123]. FGF 21 is produced mainly by the liver and secreted by BAT upon thermogenic activation. Its effects include favoring glucose homeostasis, reducing hyperlipidemia, and preventing obesity [123]. Released into circulation by BAT, circulating miRNAs, including exosomal miRNA-99b, have been proposed to act on the liver, exerting control over hepatic FGF21 expression [124].

Interleukin 6 (IL6) is a proinflammatory cytokine and myokine released by the skeletal muscle during exercise [125,126]. It has also been demonstrated that IL6 is secreted by BAT [125]. IL6 promotes pancreatic insulin secretion [127], improves glucose metabolism in adipocytes and the liver, and maintains thermogenesis [128].

Another notable batokine, 12,13-diHOME, has recently been identified for its beneficial roles in cardiovascular function [66]. In a BAT transplantation model, it was observed that 12,13-diHOME released from BAT significantly enhanced cardiac function, as evidenced by improved left ventricular hemodynamics and enhanced cardiomyocyte respiration through heightened calcium cycling. Furthermore, increases in 12,13-diHOME were observed not only with BAT transplantation but also in response to both cold exposure and exercise training [60,67]. Additionally, BAT secretes a myriad of other lipids with diverse physiological effects, including omega-3 fatty acids, oxylipins, and other lipid mediators [129,130,131]. These lipids contribute to various metabolic processes, such as thermogenesis, glucose uptake, and inflammation regulation, underscoring the multifaceted role of BAT in metabolic homeostasis.

Growth Differentiation Factor 15 (GDF15) is another batokine [125]. In healthy individuals, GDF-15 is mainly expressed in the placenta, followed by the prostate, although low levels of expression have been detected in different organs [132]. The effects of GDF-15 are pleiotropic and include appetite regulation and effects on metabolism, pregnancy, cell survival, immune response, and inflammation [132].

Neuregulin4 (NRG4) is an epidermal growth factor-like protein secreted by BAT that represses hepatic lipogenesis [133]. NRG4 increases hepatic fatty acid oxidation, inhibits de novo lipogenesis, and, therefore, potentially protects from nonalcoholic fatty liver disease (NAFLD) [133]. Transgenic overexpression of NRG4 in mice fed a high-fat diet resulted in decreased weight gain and improved glucose tolerance and insulin sensitivity [133].

C-X-C motif chemokine ligand-14 (CXCL14) is another cytokine that promotes the recruitment of M2-type macrophages [134]. M2 macrophages are associated with anti-inflammatory responses and are often linked to tissue repair and remodeling. Therefore, the release of CXCL14 during thermogenic activation may contribute to the establishment of a more anti-inflammatory adipose tissue environment, supporting the beneficial metabolic effects associated with BAT activity [134]. A lack of CXCL14 impairs brown fat activity and alters glucose homeostasis [134]

Through the secretion of these batokines, BAT establishes connections with other organs, contributing to essential physiological functions in the regulation of adaptive thermogenesis, lipid and glucose homeostasis, and metabolic crosstalk to modulate systemic metabolism.

## 12. Circadian Rhythm and Brown Fat Activation

Circadian rhythms regulate many biological processes in response to environmental influences. An altered circadian rhythm is associated with obesity and obesity-related metabolic disorders. BAT may play an important role in this process as it has a high capacity to burn fat and release energy as heat, supporting the fight against obesity and its associated metabolic disorders [135].

Animal studies suggest that UCP1 gene expression in BAT is rhythmic over 24 h and that thermogenesis has a circadian rhythm, leading to circadian body temperature [136]. Glucose uptake by BAT is rhythmic, as observed by PET/CT imaging [137]. Fatty acid synthesis demonstrates the presence of circadian rhythm in BAT in mice [138].

In addition to animal studies, human studies have demonstrated the presence of circadian rhythm in BAT [13]. In both mice and humans, the uptake of fatty acids by BAT shows rhythmicity, which may explain fluctuations in plasma lipid concentrations [139].

Circadian disruption contributes to obesity and related metabolic disorders. The fact that BAT also functions according to the circadian rhythm means that circadian rhythm disturbances (e.g., shift work) can also be caused by damage to BAT.

## 13. Browning of WAT: Implications for Metabolic Health and Pathological Conditions

The browning phenomenon is viewed positively, as it has the potential to address metabolic imbalances caused by a hypercaloric diet, thereby enhancing the lipid and carbohydrate profiles of individuals with obesity and facilitating weight loss. However, this may not be possible without side effects. As shown in Table 2, various BAT promotion agents also exhibit side effects.

Exploring the transformation of white fat into brown fat and its potential impact on promoting leanness and increased metabolic activity, recent studies have implicated browning of WAT as a potential factor in the development and progression of hypermetabolism associated with cachexia [140,141]

Cachexia is a metabolic wasting syndrome characterized by severe weight loss, systemic inflammation, and atrophy of the WAT and skeletal muscle. It is most commonly observed in cancer patients but has also been associated with burn injuries, infectious diseases (HIV, tuberculosis), and chronic diseases [92]. Cachexia contributes to a poor prognosis in these patients. Browning of WAT under pathological conditions, such as cancer and burn injury, adds fuel to an already highly catabolic state. Cytokines and tumor-secreted factors may be responsible for inducing browning of the subcutaneous white adipose tissue. IL6 is a cytokine that has been shown to induce and sustain browning of WAT in cachexia [140], and blocking it with a neutralizing monoclonal antibody or nonsteroidal anti-inflammatory drug reduces the severity of cachexia and suppresses the browning capacity of subcutaneous WAT [140].

## 14. Conclusions

In light of the increasing global health concerns caused by the obesity epidemic, it is crucial to prioritize the pursuit of effective treatments and preventive strategies. BAT, with its remarkable thermogenic capacity and affinity for glucose and fatty acids, emerges as a promising therapeutic target for inducing weight loss and enhancing blood sugar and lipid control. The current challenge lies in identifying specific molecules or compounds that selectively target these adipocytes, offering metabolic benefits such as increased fat oxidation, reduced body fat, and improved glucose homeostasis, all while minimizing side effects.

Some of the most promising molecular targets for promoting BAT thermogenesis include GLP1 and FGF-21. However, a comprehensive understanding of their broader impact on metabolism and physiology requires further investigation. Notably, the literature consistently highlights the activation of BAT in response to reduced ambient temperature, although the practical implementation of cold therapy poses logistical challenges that warrant further exploration.

In our contemporary society, where obesity is on the rise and existing drugs show limited long-term success, the discovery of BAT has opened new avenues for combating this prevalent issue. BAT’s active role in lipid and glucose metabolism positions it as a valuable therapeutic tool against obesity and related diseases. Ongoing research explores innovative areas, including exosome-mediated delivery of miRNAs, pharmacological activation of BAT thermogenic activity, and modulation of specific batokines.

Although BAT activation has been shown to have beneficial effects on carbohydrate and lipid profiles, concerns about potential side effects persist. Substances known to promote BAT activation have been associated with side effects, notably an increased risk of oncogenesis and cardiovascular system overstimulation. Ongoing research is being conducted to fully comprehend and effectively address the possible downsides. Despite the substantial progress in unraveling BAT biology over the past decade, translating these findings into clinically significant treatments for metabolic diseases remains a key frontier. 

## Figures and Tables

**Table 1:** White, beige, and brown fat cells.

Characteristics	White Fat Cell	Beige Fat Cell	Brown Fat Cell	References
	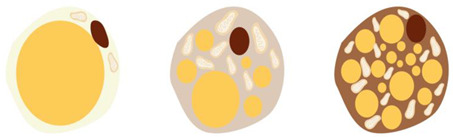			[1]
Location	Subcutaneous, visceral	Supraclavicular, predominantly dispersed in white adipose tissue	Interscapulovertebral (infants), cervical, supraclavicular, axillary, perirenal, paravertebral, around arteries and organs	
Color	White	Beige	Brown	
Proportion of mitochondria	Low	Medium	High	
Lipid drops	Unilocular, occupying approximately the entire cell	Multilocular, small	Multilocular, small	
UCP1 expression	Low/undetectable	Medium	High	
Thermogenetic activity	Low	Medium	High	
Role	Triglyceride storage, endocrine organ	Thermogenesis, endocrine organ	Thermogenesis, endocrine organ	

**Table 2:** Summary of substances and methods to activate brown adipose tissue.

BAT Promotion Agents	Activation Method	Additional Effects	Possible Side Effects	References
1. Cold exposure	Exposure to cold temperatures increases sympathetic nervous system activity, releasing norepinephrine that acts on β3 receptors in BAT.	Treats depression Releases endorphins	Aggravates hypertension Discomfort during exposure Potentially increased frequency of respiratory infections	[10,37,38,39,40,41,42,43,44,45,46,47]
2. Mirabegron	β3 agonist mediating thermogenesis in BAT and lipolysis in white adipose tissue.	Used to treat overactive bladder	Increased heart and pulse rate Negative cardiovascular effects at high doses	[16,50,51,52]
3. Physical exercise	Increases sympathetic nervous system activity, secretion of irisin, FGF21, and cardiac natriuretic peptides.	Improves insulin sensitivity Glucose tolerance Reduces circulating lipids	Side effects can occur when exercise is associated with certain pathologies (severe hypertension, fever, coronary heart disease)	[53,54,55,56,57,58,59]
4. 12,13-diHOME	Lipokine secreted by BAT under cold exposure and exercise regulates BAT fuel uptake.	Negative association with BMI and triglyceride levels Increases cardiac function	No significant adverse effects were reported	[60,65,66,67,68]
5. Capsaicinoids	Ligands for TRPV1 receptors stimulate the central nervous system to release noradrenaline.	Analgesia Protection from cardiovascular diseases Insulin sensitivity improvement. Anti-cancer activity (lung, prostate, breast)	Co-carcinogenic effects in skin cancer, further studies needed for alternative mechanisms Produce thermal and burning sensations upon contact	[47,70,71,72]
6. Green tea catechin	Interaction between catechin polyphenols and caffeine, affecting sympathetic norepinephrine release.	Reduces blood pressure, Improves glycemic metabolism Weight regulation Anti-cancer activity	Toxic effect on the liver at high doses	[73,74,75,76,77]
7. Fibroblast Growth Factor 21 (FGF21)	Secreted by the liver, white adipose tissue, and brown adipose tissue, it increases the expression of thermogenic genes.	Improves insulin sensitivity, Lowers blood glucose and lipid levels	Bone loss Fertility issues	[78,79,80,81,82,83,84,85,86,87]
8. Vascular Endothelial Growth Factor A (VEGFA)	Angiogenetic growth factor acts in a paracrine manner to modulate vascularization and activate thermogenesis in BAT.	Promotes angiogenesis	Limited due to anti-VEGF therapies applied in cancer treatment	[38,88,89,90,91,92]
9. Thiazolidinediones (TZDs)	PPAR-γ agonists induce thermogenesis-specific gene expression.	Insulin-sensitizing function	Heart failure Edema Weight gain Bone loss	[92,93,94,95,96]
10. Thyroid hormones	Key regulators of metabolism induce WAT browning.	Influences growth, lung, heart function, skeletal muscle development	Heart problems Hyperthermia Weight loss	[97,98,99,100,101,102]
11. Bone Morphogenetic Protein-7 (BMP-7)	Promotes differentiation of mesenchymal progenitor cells to a brown adipocyte lineage.	Reduces fat mass Lower plasma glucose Bone formation	Swelling Seroma Increased cancer risk	[34,103,104,105,106]
12. GLP-1R agonists	Upregulate UCP-1 protein levels in BAT and increase insulin secretion.	Slow gastric emptying, Decreases appetite	Nausea Vomiting Diarrhea	[107,108,109,110,111]

## Data Availability

Not applicable.

## Abbreviations

12,13-diHOME	12,13-dihydroxy-9Z-octadecenoic acid
BAT	Brown adipose tissue
cAMP	Cyclic Adenosine Monophosphate
CIDEA	Cell death-inducing DFFA-like effector A
CREB	cAMP response element-binding protein
CXCL14	C-X-C motif chemokine ligand-14
DIO2	Stromal iodothyronine deiodinase 2
EPA	Eicosapentaenoic acid
FGF 21	Fibroblast Growth Factor 21
FDG	2-18F-fluoro-2-deoxy-glucose
GDF15	Growth Differentiation Factor 15
GLP-1R	Glucagon-like peptide-1 receptor
HSL	Hormone-sensitive lipase
MAPK	Mitogen-activated protein kinase
Myf5	Myogenic factor 5
NRG4	Neuregulin4
PET/CT	Positron emission tomography–computed tomography
PGC1α	Peroxisome proliferator-activated receptor-gamma coactivator
PKA	Protein kinase A
PPAR-γ	Peroxisome proliferator-activated receptor γ
PRDM16	PR Domain-Containing 16
SNS	Sympathetic nervous system
TRPV1	Transient Receptor Potential Vanilloid channel 1
TMEM26	Transmembrane protein 26
UPC1	Uncoupling Protein 1
WAT	White adipose tissue
